# Dry-Cured Ham-Derived Peptide (Asp–Leu–Glu–Glu) Exerts Cytoprotective Capacity in Human Intestinal Epithelial Caco-2 Cells

**DOI:** 10.3390/antiox10091354

**Published:** 2021-08-26

**Authors:** Lujuan Xing, Lijuan Fu, Yuejing Hao, Wangang Zhang

**Affiliations:** Key Lab of Meat Processing and Quality Control, Jiangsu Collaborative Innovation Center of Meat Production and Processing, College of Food Science and Technology, Nanjing Agricultural University, Nanjing 210095, China; lujuanxing@njau.edu.cn (L.X.); 2019108068@njau.edu.cn (L.F.); 9171810323@njau.edu.cn (Y.H.)

**Keywords:** bioactive peptide, dry-cured ham, Asp–Leu–Glu–Glu, antioxidant enzymes, Nfr2/Keap1

## Abstract

Dry-cured hams are well-known and highly appreciated products in the Mediterranean and China. The long-term fermentation endows dry-cured hams with a unique flavor and quality. Our previous study has identified Asp–Leu–Glu–Glu (DLEE) from dry-cured Xuanwei ham with remarkable antioxidant capacity. In the current study, the Caco-2 cells were cultured in vitro and treated with different doses of DLEE. The cellular reactive oxygen species (ROS) level and antioxidant enzyme activities were then determined to investigate the intracellular protection effect of DLEE. According to the results, the cellular ROS level was reduced, whereas the antioxidant enzyme activities of glutathione reductase, catalase, and glutathione peroxidase were improved following DLEE treatment. The DLEE treatment also increased the Nrf2 expression, along with downregulating the Keap1 expression. Thus, the dry-cured ham-derived peptide DLEE exhibited excellent bioactive capacity by reducing the ROS level and regulating the antioxidant enzyme activities. In addition, Nrf2/Keap1 was shown to be the main signaling pathway underlying DLEE-induced antioxidant activities in Caco-2 cells.

## 1. Introduction

Reactive oxygen species constitute the superoxide anion (O_2_^•−^), hydrogen peroxide (H_2_O_2_), hydroxyl radical (^•^OH), lipid peroxide (LO^•^, LOO^•^, LOOH), and other molecules with strong oxidative capability [1]. ROS are the byproducts of oxygen metabolism and possess unpaired electrons that trigger free-radical chain reactions. Excess ROS accumulation in organisms can disturb the redox balance and destroy the structure of nucleic acids and proteins. On the other hand, lipid peroxidation, amino-acid oxidation in proteins, DNA modification, and the activation of nuclear transcription factors constitute the major processes that decrease the ROS level, which can lead to a series of diseases, including chronic inflammatory syndrome, Alzheimer’s disease, and other age-related diseases [2]. In epidemiological studies, intestinal ischemia, enteritis, and inflammatory bowel diseases are also reported to be related to the attraction of ROS [3]. In organisms, enzymatic and nonenzymatic cellular antioxidant systems play an important role in scavenging ROS. Superoxide dismutase (SOD), glutathione reductase (GR), glutathione-*S*-transferase (GST), catalase (CAT), and glutathione peroxidase (GSH-PX), are antioxidant enzyme families which can inhibit the generation of free radicals, as well as lipid hydroperoxides [4]. Ascorbic acid, glutathione (GSH), α-tocopherol, and β-carotene are nonenzymatic molecules with a significant role in defending against oxidative stress [5], among which only GSH can be produced in cells by glutathione synthetase. GSH can detoxify peroxidized lipid byproducts in GST-catalyzed reactions. Under the catalysis of GSH-PX, GSH can react with H_2_O_2_ and generate glutathione disulfide (GSSG) and H_2_O. GSSG can be restored to GSH under the catalysis of glutathione reductase (GR) in a cyclic action to protect against oxidative stress in organisms.

In order to maintain the ROS balance in vivo, food-derived antioxidants are also proposed to be involved in redox reactions, along with an alleviating effect on oxidative stress. The bioactive peptides from soybean [6], cereal flours [7], and marine [8] and meat products [9,10] have all been reported to quench superoxide free radicals, chelate metal ions, and reduce the generation of oxidative metabolites. In addition to chemical assays evaluating the antioxidant activity, it is also necessary to determine the cell-based cytoprotective effect demonstrating the regulation of oxidation, as well as explaining the peptide-induced signal pathways. Caco-2 cells have been widely used to evaluate the activity of biological and functional compositions in vitro. Fish skin gelatin [11], eggshell membrane proteins [12], and squid-derived antioxidant peptides [13] have all been shown to regulate cellular enzyme activity and exhibit a cytoprotective effect in Caco-2 cells.

Dry-cured hams are highly appreciated products with unique flavor and quality in the Mediterranean and Asian areas. During fermentation, a high extent of hydrolysis occurs, which produces large amounts of amino acids and peptides that improve the sensory quality of hams [14]. In the previous studies on Spanish and Chinese dry-cured hams, antioxidant peptides were isolated and identified with remarkable activity in scavenging DPPH, O_2_^•−^, and ^•^OH free radicals [15,16]. In particular, our research group first identified the antioxidant peptide Asp–Leu–Glu–Glu (DLEE) from dry-cured Xuanwei ham showing an excellent free-radical scavenging effect in vitro [9]. In order to further illuminate the intracellular protection effect, Caco-2 cells were cultured in vitro and treated with varying doses of DLEE. The cellular ROS level, the activity of antioxidant enzymes, and the expression of antioxidant proteins were measured to reveal the potential pathways underlying DLEE-induced antioxidant activities.

## 2. Material and Methods

### 2.1. Materials

The Caco-2 human intestinal cell line, Dulbecco’s modified Eagle medium (DMEM), penicillin–streptomycin, and trypsin-EDTA (0.25%) were purchased from Jiangsu KeyGen BioTech (Nanjing, China). Hank’s balanced salt solution (HBSS) and fetal bovine serum (FBS) were purchased from Gibco (NY, USA). CAT, GSH-PX, and GR antioxidant enzyme kits were purchased from Nanjing Jiancheng Bioengineering Institute. The primers for real-time PCR were synthesized by Genscript Biotechnology Co., Ltd. (Nanjing, China). Total RNA, and cDNA synthesis kits were purchased from Takara Bio. Inc. The peptide fraction of DLEE (98% purity) was synthesized by China Peptides Co. (Suzhou, China).

### 2.2. Cell Culture

Caco-2 cells were cultured in DMEM, and supplemented with 10% FBS and 1% penicillin–streptomycin. The incubator was kept at 37 °C containing 5% CO_2_, and the medium was changed every 2 d until the confluence reached 80%. After being dispersed by trypsin-EDTA (0.25%), the cells were cultivated in plates for further use.

### 2.3. Cellular Antioxidant Activity

The cellular antioxidant activity (CAA) of Caco-2 cells was determined following treatment with DLEE and GSH according to the study by Torres et al. [17]. Firstly, the Caco-2 cells were incubated in a 96-well microplate with a density of 6 × 10^4^ cells/well. DMEM with 10% FBS was used to culture the cells for 48 h. Then, the cells were treated with 0.5, 1.0, or 1.5 mg/mL DLEE and GSH for 2 h, followed by adding 25 μM DCFH-DA for 1 h. Then, the DMEM medium was replaced by PBS to wash the cells, along with 100 μL of 25 μM 2′,7′-dichlorodihydrofluorescein diacetate (DCFH-DA) in DMEM with 5% FBS to be incubated for another 1 h. After washing with PBS again, ABAP (600 mM) was applied as the radical generator. Lastly, the 96-well microplate was quickly analyzed using a fluorescence microplate reader (Spectral Max M2e, Sunnyvale, CA, USA) at 37 °C. The emission and excitation wavelengths were 538 and 485 nm, respectively, and the measurements were taken every 5 min for 1 h. In the control plate, the cells were treated with DCFH-DA and ABAP together. In the peptide-treated plate, the cells were incubated with DCFH-DA, ABAP, DLEE, or GSH (0.5, 1.0, or 1.5 mg/mL respectively). The CAA value was calculated as follows:

CAA = 100 − (ʃSA/ʃCA) × 100,

where ʃSA is the integrated area of the sample curve, and ʃCA is the integrated area of the control curve. All tests were operated in triplicate, and data were analyzed using GraphPad Prism 5 software.

### 2.4. ROS in Caco-2 Cells

The amount of ROS in Caco-2 cells was investigated using the DCFH-DA method [18]. Caco-2 cells were incubated in 24-well plates with a density of 1 × 10^5^ cells/well. When the density was 80%, DLEE and GSH were added at a concentration of 1.0 mg/mL. After pretreatment for 2 h, the plates were washed with PBS (1×, pH 7.4), and H_2_O_2_ (1 mM) was added for an additional 2 h incubation at 37 °C. After washing with PBS, the plates were treated with DCFH-DA (10 μM) for another 30 min. Lastly, the Caco-2 cells were collected using trypsin hydrolysis (0.25%, 3 min) and washed twice with PBS. Intracellular levels of ROS were measured using a Leica DMI 6000 B with a fluorescent channel, and then the cells were placed into a black 96-well plate for the detection of the fluorescence index using a Multimode Reader. The emission and excitation wavelengths were 535 and 485 nm, respectively.

### 2.5. Oxidative Stress Treatment

Oxidative stress of Caco-2 cells was achieved following the method of Shi et al. [19] with slight modifications. Briefly, Caco-2 cells were incubated with a density of 1 × 10^5^ cells/well in 24-well culture plates. After 48 h, the medium was replaced by HBSS. In the positive control (PC), H_2_O_2_ was added to cultivate Caco-2 cells for 6 h at 37 °C. In the treatment group, the cells were incubated with DLEE (0.5, 1.0, or 1.5 mg/mL) for 2 h in advance, and then H_2_O_2_ was added for an additional 6 h. Then, the cells were washed twice and suspended in PBS (containing 1 mM EDTA) along with ultrasonic treatment (5 min with 2 s intervals) to obtain the cell lysate. Lastly, the supernatant was collected and stored at −80 °C for further use. In each plate, the negative control (NC) received no stimulation, whereas the treatment with H_2_O_2_ was regarded as the PC.

#### 2.5.1. Antioxidant Enzyme Activity

The activities of CAT, GSH-PX, and GR antioxidant enzymes were measured using the respective assay kits (Nanjing Jiancheng Bioengineering Institute) according to the manufacturer’s protocol. In brief, Caco-2 cells were separated into NC, PC, DLEE, and GSH groups. Following the process described in Section 2.5, the different groups of cells were washed and then placed into tubes with sample buffer (hydroxyethyl piperazine ethylsulfonic acid, 20 mM). The ultrasonic treatment was applied to each sample for 3 min with 5 s intervals. After centrifuging at 10,000× *g*, the supernatant was collected to measure the protein content using the BCA protein assay kit (Thermo Fisher Scientific, Shanghai, China). The enzyme activities were expressed as milliunits per mg of protein (mU/mg protein).

#### 2.5.2. RNA Isolation and PCR Array Analysis

For PCR analysis, the cells were divided into NC, PC, and DLEE treatment groups. Total RNA was isolated using the Total RNA Kit according to the manufacturer’s instructions (Omega Laboratories, Inc., GA, USA). The cDNA was reverse-transcribed using a Prime Script™ cDNA Synthesis Kit (Takara, Kyoto, Japan) and quantified using a NanoDrop^TM^8000 (Thermo Fisher Scientific) with Premix EX Taq™ (Takara, Kyoto, Japan). The RT-PCR process was carried out with the following conditions: denaturation for 15 s at 95 °C, annealing for 15 s at 56 °C, and extension for 30 s at 72 °C. The gene expressions were calculated using the ∆∆Ct method with glyceraldehydes-3-phosphate dehydrogenase (*GAPDH*) as the reference gene. Primer sequences were synthesized by Genscript as shown in Supplementary Table S1, and the fold changes were calculated with respect to the control group.

#### 2.5.3. Western Blotting

Firstly, the cells were lysed in RIPA buffer (protease inhibitor cocktail), and then the supernatant was collected to quantify the protein content using the BCA protein assay kit (Thermo Fisher Scientific). Secondly, the lysate was mixed with 2× loading buffer before boiling at 95 °C for 5 min. The running process followed the protocol of Liu et al. [20]. The 20 µg samples and standard proteins (Bio-Rad, Hercules, CA, USA) were loaded on a polyacrylamide gel (4–10%) to run for 120 min (110 V, 4 °C). Then, the proteins were transferred onto a polyvinylidene difluoride membrane within 90 min (120 V, 4 °C). The membrane was then blocked in bovine serum albumin (5%, dissolved in Tris-buffered saline and Tween-20, TBST) for 1 h at room temperature. Next, the blocked membrane was incubated with the primary antibodies Nrf2 (#12721, diluted 1:1000 in 1× TBST), Keap1 (#8047, diluted 1:1000 in 1× TBST), and GAPDH (#5174, diluted 1:2000 in 1× TBST) overnight at 4 °C. After washing with 1× TBST, the membrane was incubated with anti-rabbit IgG (HRP-linked Antibody #7074) for an additional 1 h. The antibodies were all purchased from Cell Signal Technology. Lastly, the immunocomplexes were measured using a chemiluminescence system (Thermo Fisher Scientific, Rockford, IL, USA), and the bands were analyzed using the Quantity One system (Version 4.6.2). Using the expression of GAPDH as a reference, the expressions of Keap1 and Nrf2 were calculated as a function of the intensity of each band.

### 2.6. Molecular Docking

The molecular docking between DLEE and Keap1 proteins was predicted by Discovery Studio 2016. Using CHARMM, the docking program implements soft-core monitoring and optional grid representation to dock the ligand molecule in the active site of the receptor. The high-temperature dynamics method was used to randomly search through small-molecule conformations, and then the simulated annealing method was used to optimize each conformation in the region of the receptor active site. Firstly, the small molecule ligand DLEE was imported into the program and optimized by hydrogenation, followed by applying the Charmm force field and Momany–Rone charges. Secondly, the protein data bank (PDB) number of objective protein was retrieved in https://www.rcsb.org/ (accessed on 25 March 2021). The entire PDB files of Keap1 protein were compared and finally, the 2FLU was selected with the reflecting of Kelch region on its structure. In 2FLU, the active site between Keap1 and Nrf2 protein was exposed and then the docking process was operated based on the modification of objective files. In the CDOCKER module, the maximum number of collisions in each docking test was set to 10, and then the root means square deviation (RMSD) value of every docking process was calculated. Finally, the smallest RMSD value was selected as the most the strongest combination.

### 2.7. Statistical Analysis

Statistical analyses were carried out using GraphPad Prism 7.0, and the statistical significance of the data was evaluated by one-way ANOVA and *t*-test. Results were expressed as the mean ± SD with *p* < 0.05 representing a significant difference.

## 3. Results and Discussion

In the current study, the ORAC, ABTS^+^, DPPH, and superoxide free-radical scavenging capacities of DLEE were measured in comparison with GSH. As presented in Table 1, DLEE presented scavenging effects on ABTS^+^ (148.34 µmol TE/g), showing no significant differences with GSH (157.98 µmol TE/g) using Trolox as the equivalent. The ORAC value of DLEE was 1031.54 µmol TE/g, which was lower than that of GSH (1588.84 µmol TE/g, *p* < 0.05). The same tendency was also revealed when superoxide (65.06% vs. 56.05%) and DPPH free radicals (70.53% vs. 83.25%) were scavenged by DLEE vs. GSH, respectively. According to the above results, DLEE exhibited remarkable antioxidant activity through free-radical scavenging.

### 3.1. Cellular Antioxidant Activity of DLEE in Caco-2 Cells

**Figure 1:**
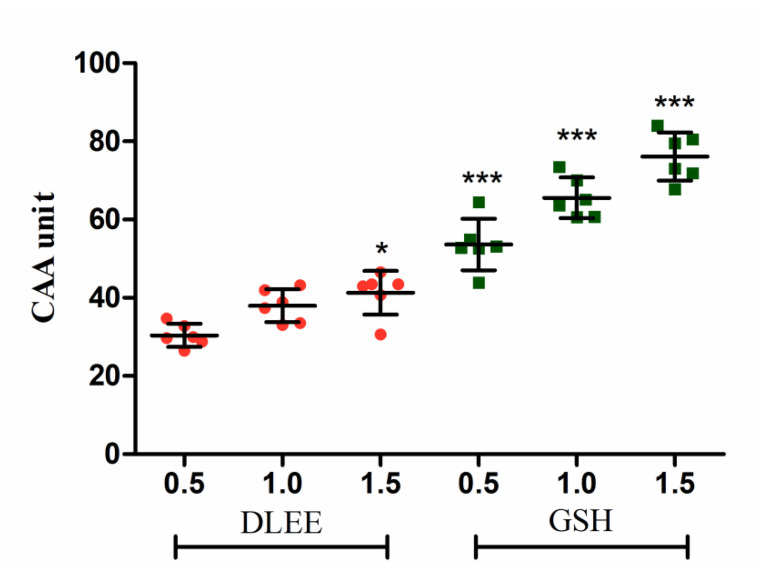
The cellular antioxidant activity (CAA) in Caco-2 cells. Cells were treated with 0.5, 1.0, or 1.5 mg/mL of peptides for 2 h, followed by 25 μM DCFH-DA for 1 h. ABAP (600 μM) was added as a radical generator. Data are presented as the means ± SD (*n* = 6). * shows the significant differences of *p* < 0.05 and *** shows the significant differences of *p* < 0.001 with DLEE-0.5 as a control in the unpaired *t*-test.

Generally, free-radical testing is commonly used for the antioxidant evaluation of bioactive components. The DCFH-DA method is typically applied in intracellular trials to reflect antioxidant capacity in living cells [21]. As shown in Figure 1, the CAA of DLEE was evaluated at concentrations of 0.5, 1.0, and 1.5 mg/mL in comparison with GSH. The cellular radical scavenging activities of DLEE and GSH were enhanced slightly with an increase in concentration. Compared with 0.5 mg/mL of DLEE, the CAA showed to be enhanced in 1.5 mg/mL DLEE treatments (*p <* 0.05). At the concentration of 0.5 mg/mL, the CAA of DLEE was calculated as 30.41, which is similar to the effect of protein hydrolysates from the eggshell membrane [22]. The CAA of DLEE peptide suggested similar free-radical scavenging activity to GSH in an intracellular environment.

### 3.2. Cellular ROS Detection in Caco-2 Cells

**Figure 2:**
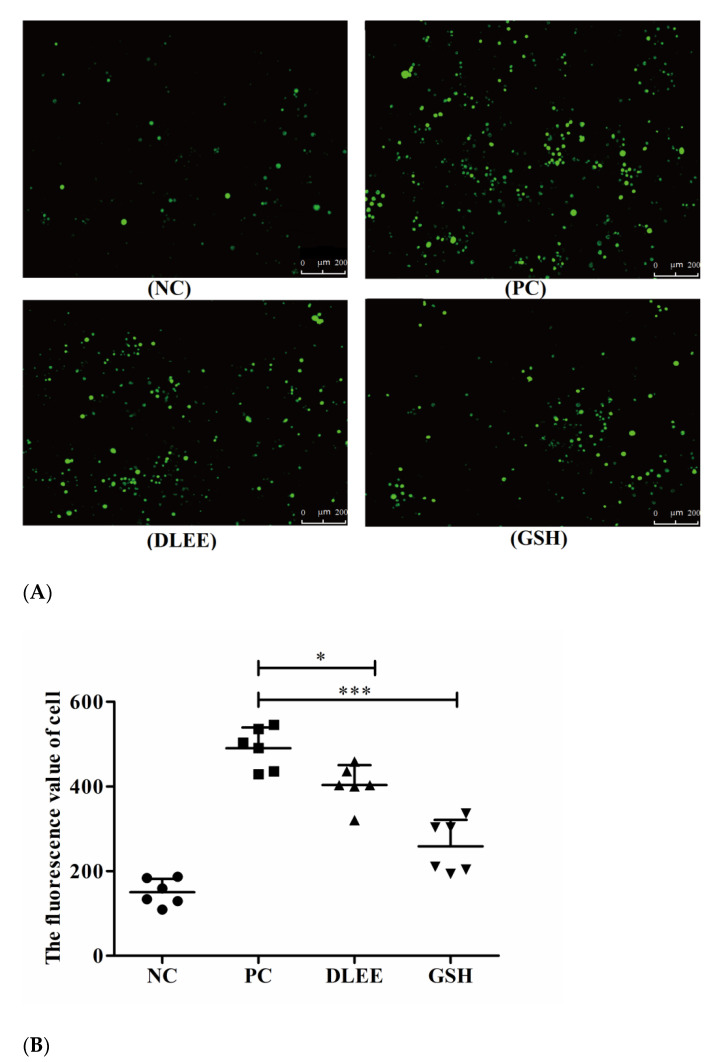
(**A**) A Fluorescence micrograph of Caco-2 cells (50×) following treatment with DLEE and GSH. (**B**) The fluorescence values of cells. NC, Caco-2 cells without H_2_O_2_; PC, H_2_O_2_-induced cells; DLEE and GSH were administered at a concentration of 1.0 mg/mL to H_2_O_2_-induced cells. Data are presented as the means ± SD (*n* = 6); * shows the significant differences of *p* < 0.05 and *** shows the significant differences of *p* < 0.001 with the unpaired *t*-test.

ROS are generated throughout the processes of cell growth, differentiation, reproduction, apoptosis, and aging, as well as due to pathological variation. Changes in the levels of ROS can also reflect the condition of cell metabolism. Figure 2 presents the fluorescent images of Caco-2 cells following treatment with 1.0 mg/mL DLEE and GSH. Compared with the control group, the fluorescence spots of the H_2_O_2_-stimulated group were significantly apparent. In the treatment groups of DLEE and GSH, the spots all presented reduced intensity. In the control group, no exogenous H_2_O_2_ was added, and the ROS fluorescence value was 150, which increased to 490 upon H_2_O_2_ stimulation, indicating that exogenous H_2_O_2_ could promote the accumulation of ROS in Caco-2 cells. Once incubated with 1.0 mg/mL DLEE in advance, the fluorescence intensity decreased from 490 (PC) to 403 (*p* < 0.05), suggesting that DLEE could actively inhibit the production of ROS in Caco-2 cells. In oxidized HepG2 cells, the intracellular ROS scavenging activity of corn gluten peptides was also evaluated using fluorescence images, where it was shown that 1.0 mg/mL of different peptide fractions all exhibited remarkable reducing effects on ROS ranging from 47.5 (oxidized group) to 22.3 (molecule weight < 1 kDa) and 23.5 (molecule weight 1–3 kDa) [23]. In addition, comparable results were also obtained in the bioactive peptides derived from Nile tilapia proteins [24]. Lima et al. [25] reported that the cellular antioxidant capacity of bioactive components relies on transmembrane transport into cells and antioxidant efficiency in cell culture conditions. Wiriyaphan et al. [26] demonstrated that threadfin bream-derived hydrolysate exhibited remarkable protective ability in HepG2 cells, which was related to a higher cell permeability of lower-molecular-weight peptides identified in the hydrolysate. Here, the molecular weight of DLEE was 504 Da, and the *P*_app_ value of transportation across the Caco-2 cell monolayer was 3.22 × 10^−6^ cm/s [27]. Thus, the intracellular capacity of DLEE may also be explained by its cell permeability in Caco-2 cells.

### 3.3. Gene Expression of Antioxidant Enzymes, Nrf2, and Keap1

**Figure 3:**
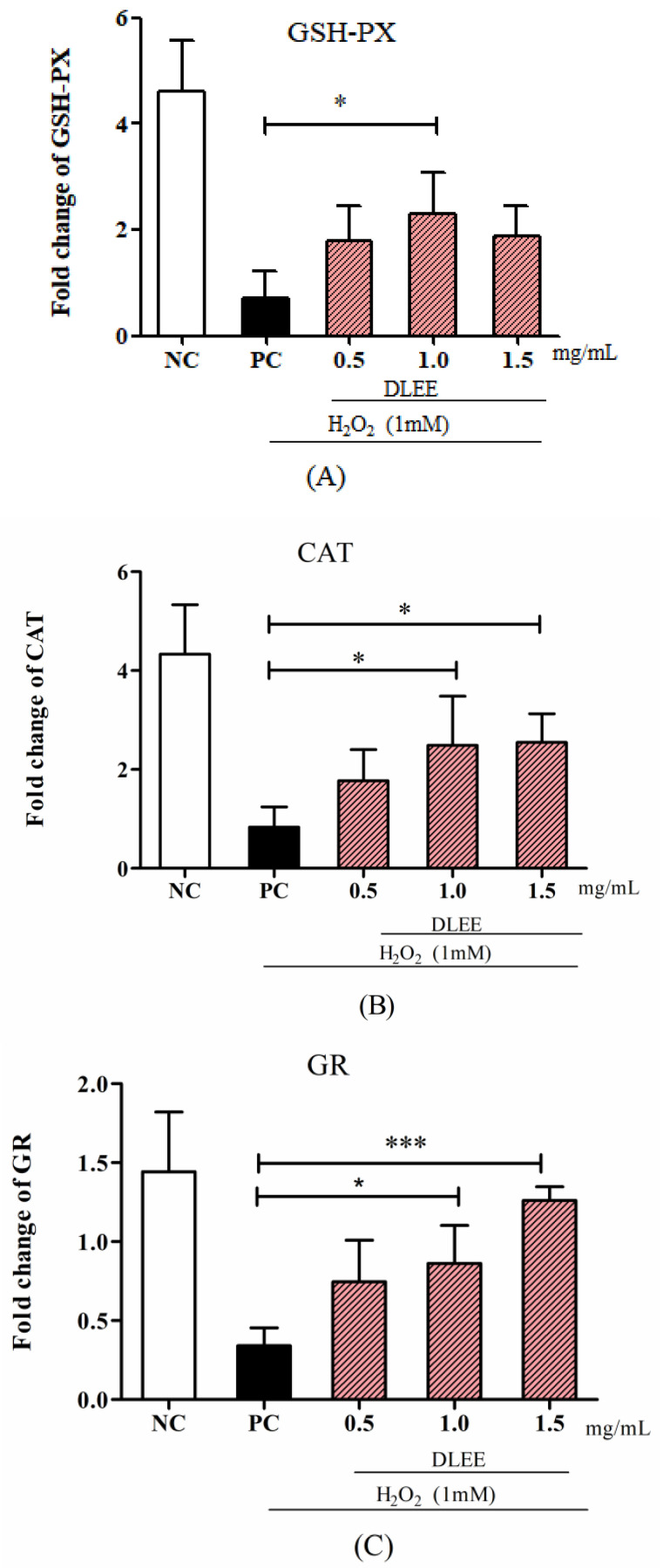

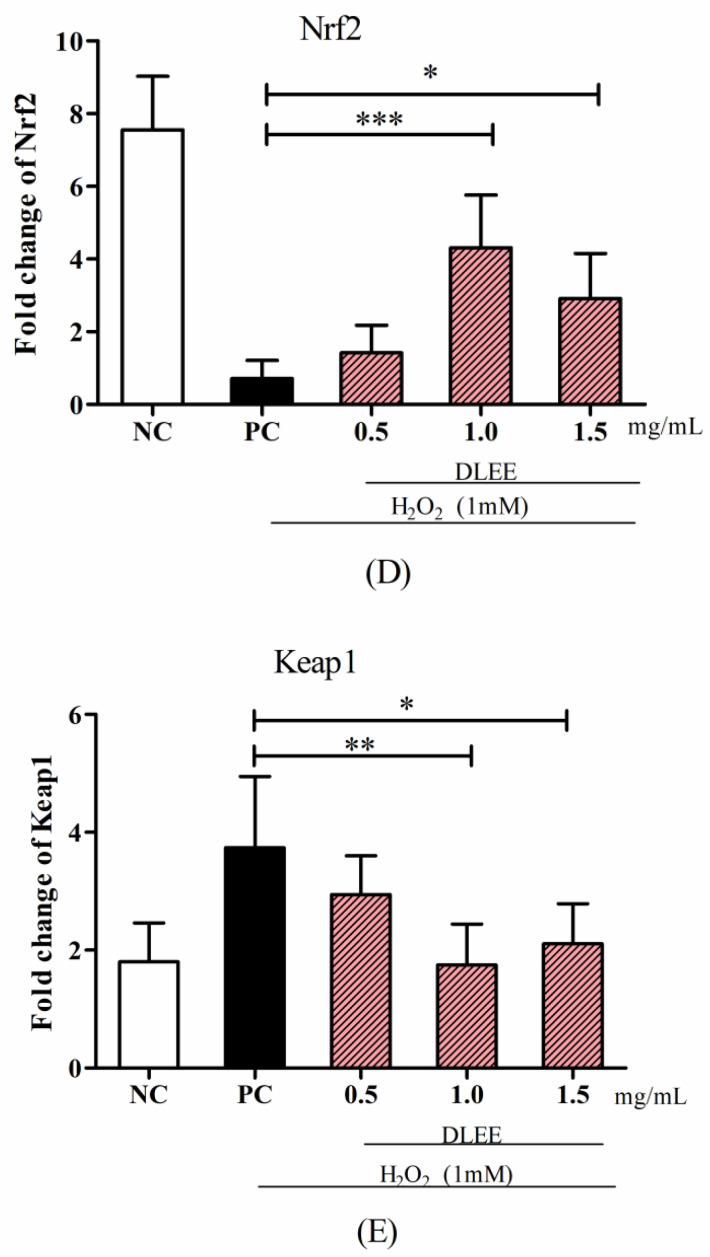
The mRNA levels of antioxidant enzymes and proteins. (**A**) GSH-PX, (**B**) CAT, (**C**) GR, (**D**) Nrf2, and (**E**) Keap1. NC, negative control group without any treatments; PC, H_2_O_2_-induced positive control group (1 mM); different concentrations of DLEE (0.5, 1.0, or 1.5 mg/mL) were added for 2 h as pretreatment along with H_2_O_2_ (1 mM). Data are presented as the means ± SD (*n* = 6); *, **, *** represent significant differences with *p* < 0.05, *p* < 0.01, *p* < 0.001, respectively.

The gene expression of antioxidant enzymes, as well as of Nrf2 and Keap1, is shown in Figure 3. Compared with NC, the expressions of GSH-PX, GR, and CAT in the PC group were significantly decreased, whereas the DLEE-pretreated group exhibited an improving effect in a dose-dependent manner. In particular, compared with PC, the cells treated with 1.0 mg/mL DLEE showed a significant increase in mRNA levels of GSH-PX and CAT (*p* < 0.05). It is also noteworthy that the highest mRNA levels of GR in the 1.5 mg/mL groups (1.45) presented remarkably significant differences with the PC group (0.43, *p* < 0.001). These results imply that DLEE may exhibit antioxidant activities by improving the expression of antioxidant enzymes in Caco-2 cells.

Meanwhile, pretreatment with DLEE may also be involved in regulating the mRNA expressions of Nrf2 and Keap1 in Caco-2 cells (Figure 3D,E). Among all groups, PC exhibited the lowest mRNA expression of Nrf2 (0.87) and the highest mRNA expression of Keap1 (3.98) without DLEE treatment. Compared with PC, the gene expression level of Nrf2 in the DLEE treatment groups was increased 3.3-fold (1.5 mg/mL) and 4.9-fold (1.0 mg/mL). These results are comparable to the effect of peptides (1.0 mg/mL) from baijiu, which increased the Nrf2 gene expression level 3.28-fold compared with the control [28]. On the contrary, compared with PC (3.87), the gene expression of Keap1 was reduced upon 1.0 mg/mL (1.87) and 1.5 mg/mL (2.17) DLEE pretreatment. Nrf2/Keap1 signaling is an important pathway for organisms to defend against oxidative stress during cell metabolism. The release of Nrf2 can upregulate the expression of downstream genes and proteins, along with the regulation of intracellular mechanisms, to promote antioxidant activities [29]. Among the Nrf2-induced downstream genes, intracellular redox-balancing proteins belong to primary antioxidant enzyme families including GSH-PX, CAT, γ-glutamylcysteine synthetase (γ-GCS), GR, peroxiredoxin, thioredoxin, heme oxygenase-1, and phase II detoxifying enzymes [30]. According to the study of Federica et al. [31], oxidative stress could stimulate Nrf2/Keap1 signaling, and the H_2_O_2_-induced expression of GR decreased in Caco-2 cells. In the rat model, the GR level also decreased in the oxidative stress group, whereas the antioxidant peptide-treated group was ameliorated. According to the results of the current study, the DLEE may be involved in the regulation of the Nrf2/Keap1 signaling pathway to protect against oxidative stress in Caco-2 cells.

### 3.4. Activities of Antioxidant Enzymes

**Figure 4:**
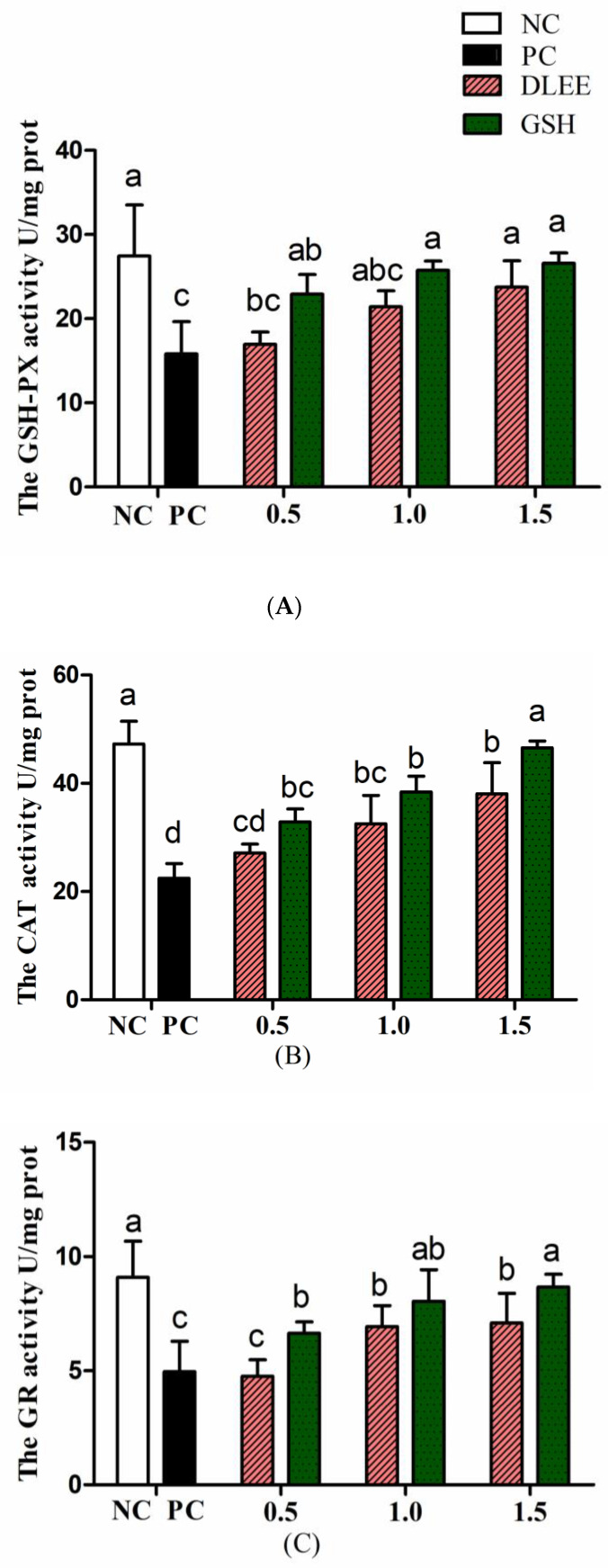
The antioxidant enzyme activities of Caco-2 cells. (**A**) GSH-PX, (**B**) CAT, (**C**) GR. NC, negative control group without any treatments; PC, H_2_O_2_-induced positive control group (1 mM, 2 h); different concentrations of DLEE and GSH (0.5, 1.0, or 1.5 mg/mL) were added for 2 h along with H_2_O_2_. Data are presented as the means ± SD (*n* = 6) and the differences between groups were compared by Tukey’s Multiple Comparison Test in a One-way analysis. The same letters represent no differences and different letters denote a significant difference between groups (*p* < 0.05).

To verify the cytoprotective effect, the antioxidant enzyme activities of Caco-2 cells with ranging concentrations of DLEE pretreatment are shown in Figure 4. Stimulated by H_2_O_2_, the activities of GSH-PX, GR, and CAT were all reduced compared with NC. After preincubating with DLEE and GSH, the enzyme activities were alleviated in a dose-dependent manner. The CAT activity increased from 32.5 U/mg (PC) to 38.7 U/mg following incubation with 1.5 mg/mL DLEE. Similarly, DLEE incubation was observed to have a positive effect on the activities of GSH-PX and GR. In cells, the antioxidant enzyme activity (CAT, GSH-PX, and GR) has an important role in scavenging free radicals, as well as defending against stimulation from foreign invasion. Shi et al. [19] evaluated the antioxidant effect of peptides isolated from egg membrane proteins. Compared with the H_2_O_2_-treated groups, the enzyme activities (including GSH-PX, GR, and GCS) were significantly improved in the peptide-treated groups. In particular, at a concentration of 0.5 mg/mL, the GSH-PX activity was two-fold higher than that in the H_2_O_2_-treated groups. However, the activity of CAT was not affected by egg membrane peptides. In the present study, pretreatment with DLEE (1.0 and 1.5 mg/mL) exhibited a favorable cytoprotective effect on Caco-2 cells under oxidative stress conditions.

### 3.5. Expression of Antioxidant Proteins

**Figure 5:**
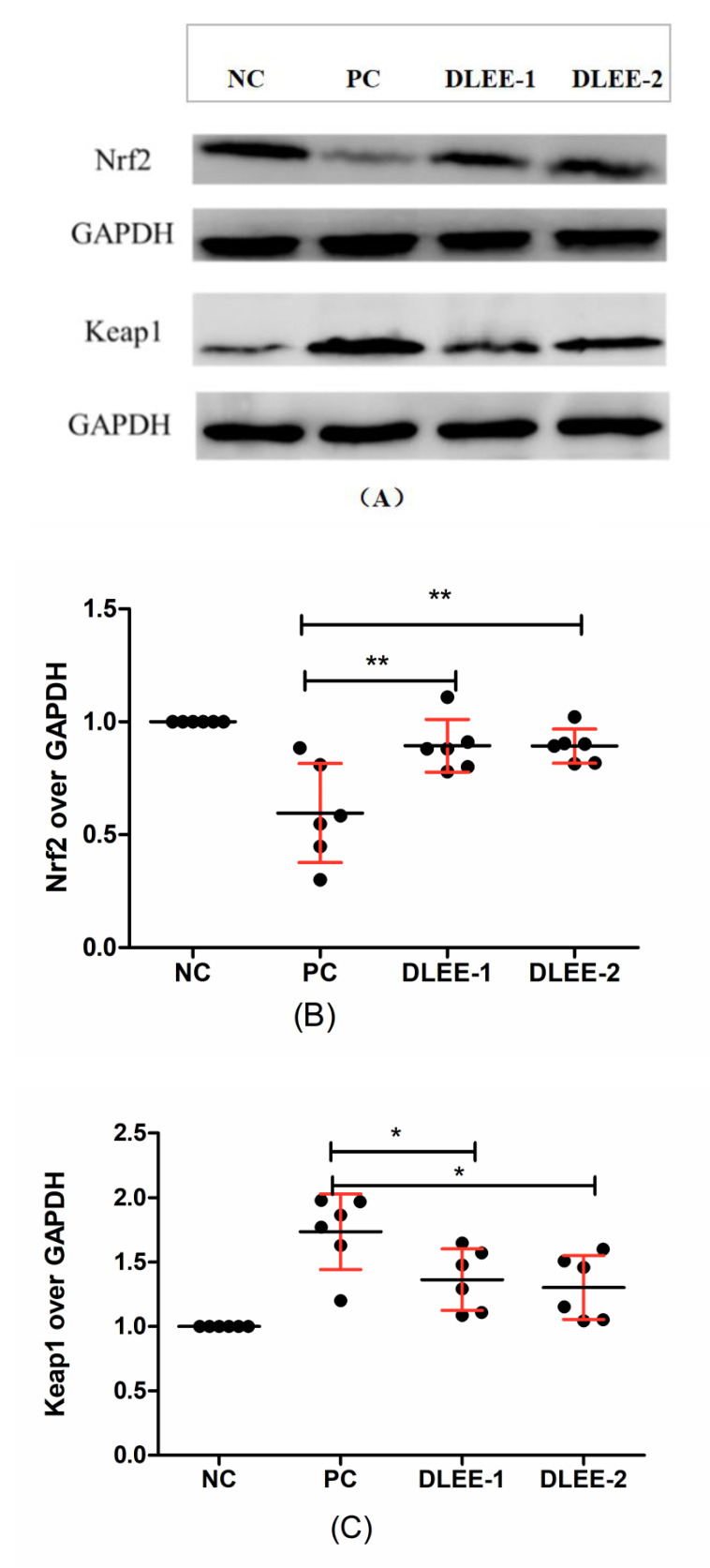
The cellular expression of Nrf2 and Keap1 in Caco-2 cells following a pretreatment with DLEE. (**A**) Nrf2 and Keap1 expressions in RAW264.7 cells; (**B**) Quantification of Nrf2; (**C**) Quantification of Keap1 expression. NC, negative control group without any treatments; PC, H_2_O_2_-induced positive control group (1 mM, 2 h); DLEE-1 and DLEE-2 represent pretreatment with 1.0 and 1.5 mg/mL DLEE (2 h) before H_2_O_2_-induced stimulation. Data are presented as the means ± SD (*n* = 6) and *, **, represent significant differences with *p* < 0.05, *p* < 0.01, respectively.

In the current study, H_2_O_2_ played the role of an oxidative stress stimulant, and the incubation of DLEE induced a potential protective effect on the cells. As shown in Figure 5, the expression of Nrf2 was downregulated in the PC group, whereas the DLEE treatment alleviated the expression of Nrf2, indicating that DLEE may have a positive effect on the expression of Nrf2 proteins. On the contrary, the expression of Keap1 was upregulated following the treatment with H_2_O_2_. This indicates that, after treatment with DLEE, the Nrf2/Keap1 signaling pathway was activated, allowing the released Nrf2 to enter the nucleus and regulate protein expression. In a previous study by Wu et al. [28], antioxidant peptides from Chinese baijiu were identified with the amino-acid sequence of DLPFKM. In Caco-2 cells, the DLPFKM-treated group reduced the expression of Nrf2, whereas Keap1 was downregulated. Following treatment with antioxidant peptides from egg proteins, the Nrf2/Keap1 signaling pathway was also activated, with the release of Nrf2 inducing the expression of antioxidant proteins and increasing the enzyme activities of CAT and SOD [32]. In the present study, the antioxidant enzyme activities of CAT, GR, and GSH-PX were also measured, which all exhibited significant differences between DLEE and H_2_O_2_ treatment groups (Figure 4). According to the results of DLEE treatment, it can be speculated that the increased activity of CAT and GR was a result of Nrf2/Keap1 pathway activation. This suggests that treatment with DLEE may effectively stimulate the release of Nrf2 from the complex, which can then enter the nucleus and regulate antioxidant enzymes and proteins.

According to a study by Majumder et al. [33], antioxidant proteins are important in maintaining cell homeostasis, and their expression levels can be affected by oxidative or inflammatory stimuli. Within the family of antioxidant proteins, Nrf2/Keap1 represents the most important signaling pathway in controlling the level of antioxidant enzymes, as well as the expression of antioxidant proteins. In homeostatic conditions, Nrf2 and Keap1 are bound together by ubiquitination, with the complex also consisting of Clu3, Rb1, and conjugating enzymes. This ligase ubiquitin complex mainly targets the Neh2 domain in Nrf2, which leads to the degradation of Nrf2 by the proteosome. However, once stimulated by ROS or another stimulant, Nrf2 is released from the complex, before entering the nuclear membrane and binding to the antioxidant response element, thereby activating the transcription of antioxidant proteins [34]. In order to further interpret the DLEE-induced process of Nrf2/Keap1 expression, molecular docking studies were carried out using Discovery Studio software.

**Figure 6:**
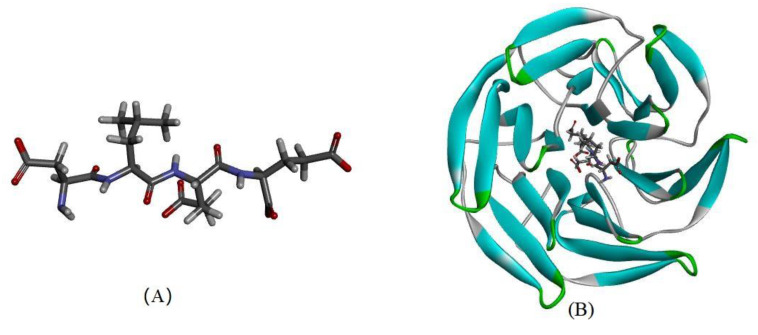

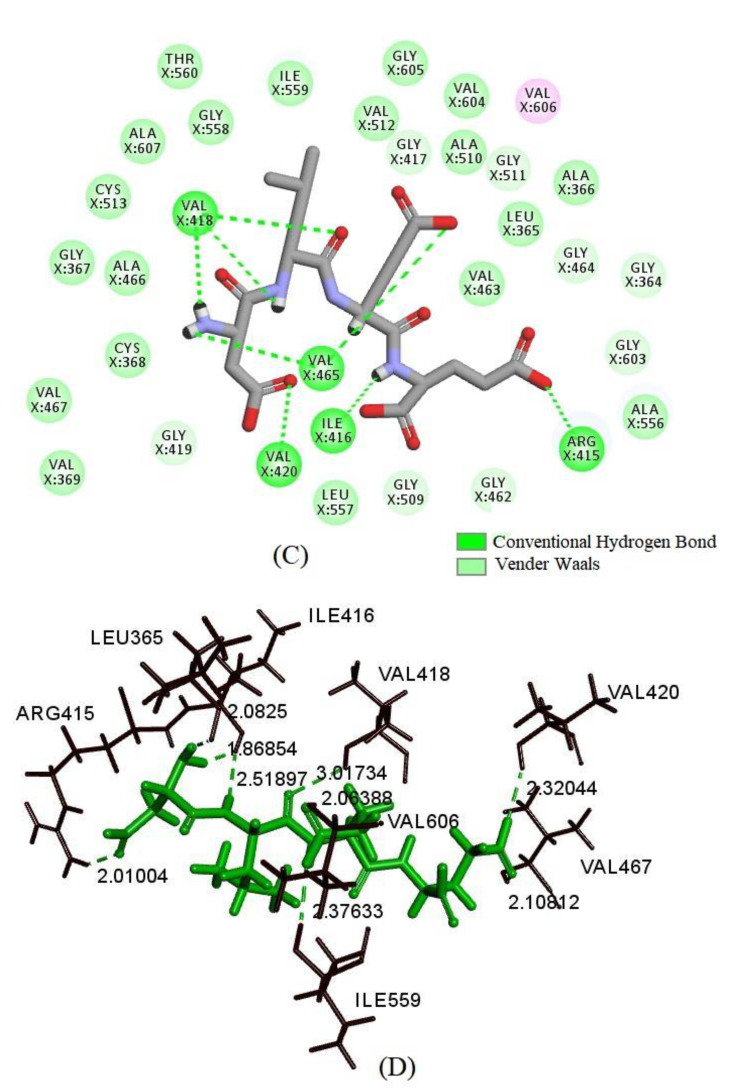
The molecular docking of DLEE peptides with Keap1 protein. (**A**) The small-molecule ligand of DLEE. (**B**) The optimal docking pose of DLEE with the Kelch domain of human Keap1 protein (PDB: 2FLU). (**C**) A 2D diagram of DLEE and Keap1 amino-acid residues. (**D**) An H-bond interaction diagram of DLEE and Keap1 amino-acid residues.

As revealed in Figure 6, DLEE was found to bind to the Kelch domain via H-bonds with an interaction energy of 92.15 kcal/mol. The most active binding site of DLEE was located in the central active pocket of the Kelch domain, involving Val418, Val 465, Ile416, Arg415, and Val420 (Figure 6C), at interaction distances of 1.89 Å, 1.95 Å, 2.32 Å, 1.91 Å, and 2.49 Å, respectively (Figure 6D). In the crystal structure analysis of Keap1 protein, the Kelch domain was the major site involved in binding to the Neh2 domain in Nrf2. The six-module β-propeller structure of the Kelch domain confers it with several active positions to interact with other protein surfaces regardless of its orientation. The Neh2 domain is located at the N-terminal of Nrf2, where the ETGE motif represents the main binding site for the Nrf2 and Keap1 interaction. In a previous study by Li et al., bioactive peptides from egg protein were also docked with the Keap1 Kelch domain (PDB: 2FLU) via hydrogen bonds. The DKK sequence interacted with Arg380 and Asn382, whereas the DDW sequence interacted with Arg415, Arg380, Asn382, Ser508, and Arg483 [32]. In addition, the DKK- and DDW-treated cells all presented higher CAT activity than the H_2_O_2_-induced injury group. Thus, despite small peptides having different biding sites on Keap1 proteins, they can all exhibit their antioxidant capacity via the Nrf2/Keap1 signal pathway. In the current study, the antioxidant enzyme activities of CAT, GR, and GSH-PX were measured, which all exhibited significant differences between the DLEE and H_2_O_2_ treatment groups (Figure 4). Accordingly, it can be speculated that the increased activities of CAT and GR were induced by Nrf2/Keap1 pathway activation. This suggests that treatment with DLEE may effectively stimulate the release of Nrf2 from the complex, before entering the nucleus and regulating antioxidant enzymes and proteins.

## 4. Conclusions

According to the intracellular antioxidant activity assays, the dry-cured ham-derived peptide DLEE was effective in reducing ROS generation and stimulating antioxidant enzyme activities. Compared with the H_2_O_2_-oxidized group, the CAT, GR, and GSH-PX gene expressions were all improved by DLEE pretreatment. In addition, the gene expression of Nrf2 was also increased, whereas that of Keap1 was decreased by DLEE incubation. The Western blot results also confirmed an identical trend for Nrf2/Keap1 protein expression. The molecular docking studies revealed that DLEE was able to bind to the Kelch domain in Keap1 with an interaction energy of 92.15 kcal/mol. Overall, DLEE was shown to have a significant effect on reducing free radicals and regulating antioxidant enzyme activities via the Nrf2/Keap1 signaling pathway in Caco-2 cells. The antioxidant capacity of DLEE needs to be further investigated in animal trials to more thoroughly evaluate its physiological regulatory activity.

## Figures and Tables

**Table 1:** The antioxidant activity of DLEE in comparison with GSH.

	DLEE	GSH
ORAC ^a^	1031.54 ± 53.87 *	1588.84 ± 141.89
ABTS^+ a^	148.34 ± 7.90	157.98 ± 8.24
Superoxide radical ^b^	65.05 ± 3.94 *	56.05 ± 3.79
DPPH radical ^b^	70.53 ± 3.91 *	83.25 ± 2.66

^a^ The synthetic DLEE and GSH were measured using Trolox as the equivalent (μmol TE/g). ^b^ The free-radical scavenging of superoxide and DPPH radicals was evaluated at a concentration of 1.0 mg/mL. Values represent the means ± SD (*n* = 5). A *t*-test was operated to determine significant differences (*).

## Data Availability

Data is contained within the article and supplementary material.

## Supplementary Materials

The following are available online at https://www.mdpi.com/article/10.3390/antiox10091354/s1, Table S1: Primer sequences.
