# Supplementary material for: Dry-Cured Ham-Derived Peptide (Asp–Leu–Glu–Glu) Exerts Cytoprotective Capacity in Human Intestinal Epithelial Caco-2 Cells
Source: Antioxidants (Basel). 2021 Aug 26;10(9):1354. doi: 10.3390/antiox10091354 (PMC8469342; doi:10.3390/antiox10091354)
Supplement: Supplementary file 1 [file antioxidants-10-01354-s001.zip › antioxidants-1304642-supplementary.pdf]

Table S1. Primer sequences

Gene	Primer	5'-primer sequences-3'
GAPDH	Forward	GGTGGTCTCCTCTGACTTCAACA
	Reverse	GTTGCTGTAGCCAAATTCGTTGC
Nrf2	Forward	AGTGTGGAGAGGTATGAGCC
	Reverse	CGTTCCTCTCTGGGTAGTAA
Keap1	Forward	AGAGCGGGATGAGTGGCA
	Reverse	GCTGAATTAAGGCGGTTTGTC
GR	Forward	AGGGCG TCA TTCACTTCG AG
	Reverse	TCCTTT CCA GCAGCCACA TT
GSH-PX	Forward	GCT CAC CCG CTC TTT ACC TT
	Reverse	GAT GTC GAT GGT GCG AAA GC
CAT	Forward	AGG CTC AGC TGA CAC AGT TC
	Reverse	GCC ATT CAT GTG CCG ATG TC
